# Supplementary material for: Long-Term Engraftment of Cryopreserved Human Neurons for In Vivo Disease Modeling in Neurodegenerative Disease
Source: Biology (Basel). 2025 Feb 19;14(2):217. doi: 10.3390/biology14020217 (PMC11852092; doi:10.3390/biology14020217)
Supplement: Supplementary file 1 [file biology-14-00217-s001.zip › biology-3471022-supplementary.pdf]

Supplementary Material

Long-Term Engraftment of Cryopreserved Human Neurons for In Vivo Disease Modeling in Neurodegenerative Disease

David J. Marmion, Peter Deng, Benjamin M. Hiller, Rachel L. Lewis, Lisa J. Harms, David L. Cameron, Jan A. Nolta, Jeffrey H. Kordower, Kyle D. Fink*, and Dustin R. Wakeman*

*** Correspondence:**
Dustin R. Wakeman, Ph.D. dwakeman@aspenneuro.com
Kyle D. Fink, Ph.D. kdfink@ucdavis.edu

1 Supplementary Figures

1.1 Supplementary Figure S1: iGABA survival at 1-week in rodent brain.

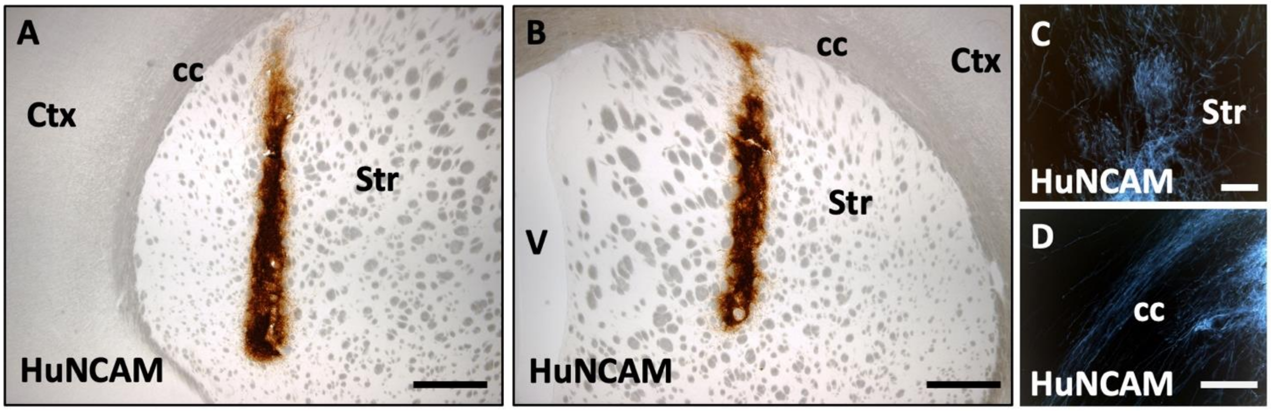

A, B. Striatal HuNCAM+ iGABA grafts in (**A**) left and (**B**) right hemispheres of NUDE rat at 1-week post-transplantation. Scale = 500 uM. **C, D**. Darkfield illumination of HuNCAM+ fibers in (**C**) striatum and (**D**) coursing through the corpus callosum. Scale = 100 uM. Abv: cc – corpus callosum, Ctx = cortex, Str = striatum, V = Ventricle.

1.2 Supplementary Figure S2: Innervation of iGABAs 9-months post-injection in the immunodeficient rat brain.

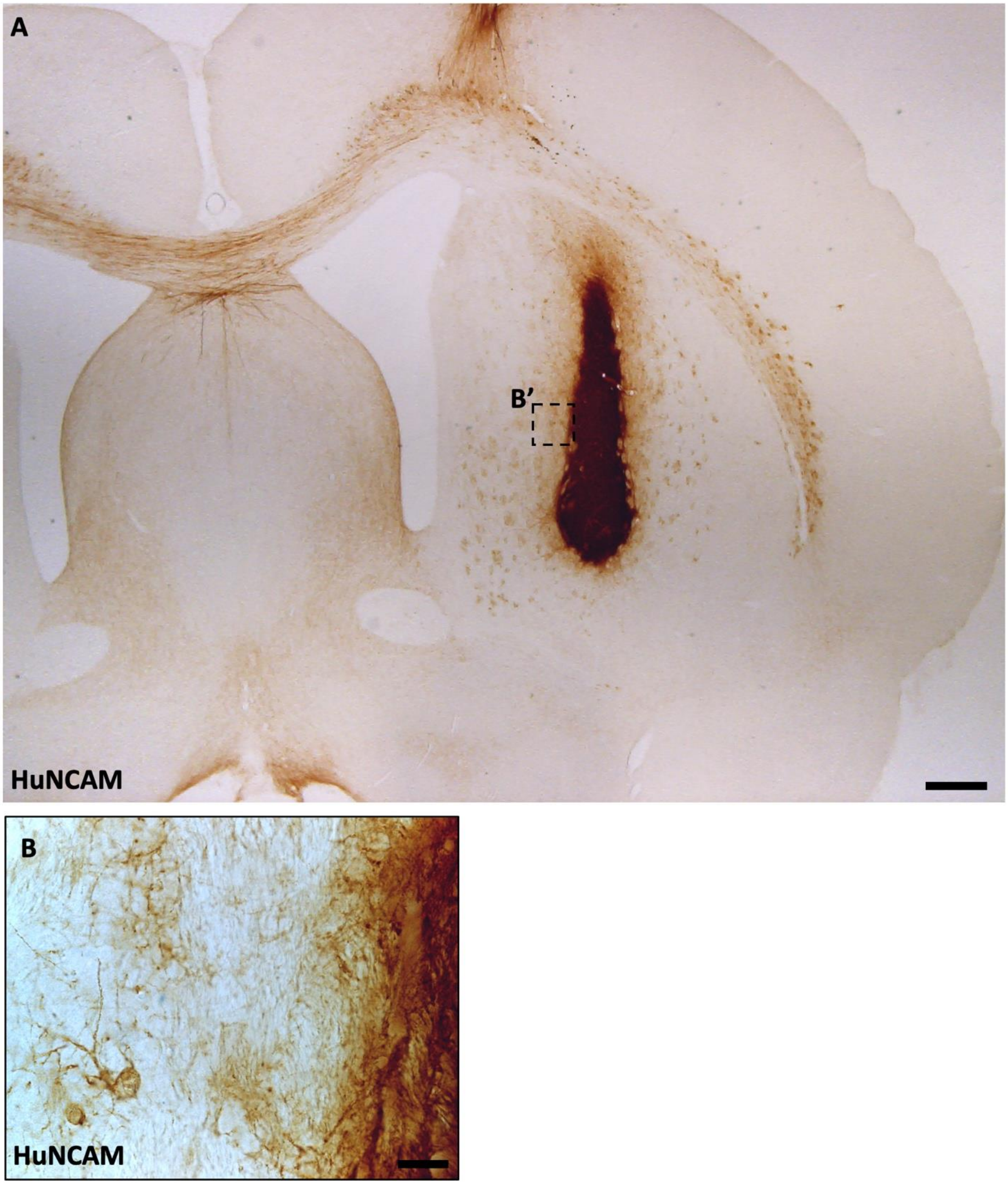

A. Widespread human NCAM staining in striatum, corpus callossum, and cortical injection zone. Scale = 500 uM. **B**. Inset of graft periphery and fiber innervation at the zone of injection. Scale = 25 uM.
